# The Large Action of Chlorpromazine: Translational and Transdisciplinary Considerations in the Face of COVID-19

**DOI:** 10.3389/fphar.2020.577678

**Published:** 2020-12-16

**Authors:** Emmanuel Stip, Tahir A. Rizvi, Farah Mustafa, Syed Javaid, Salahdein Aburuz, Nahida Nayaz Ahmed, Karim Abdel Aziz, Danilo Arnone, Aravinthan Subbarayan, Fadwa Al Mugaddam, Gulfaraz Khan

**Affiliations:** ^1^ Department of Psychiatry, University of Montréal, Montréal, QC, Canada; ^2^ Department of Psychiatry and Behavioral Science, College of Medicine and Health Science, United Arab Emirates University, Al Ain, United Arab Emirates; ^3^ Department of Medical Microbiology and Immunology, College of Medicine and Health Science, United Arab Emirates University, Al Ain, United Arab Emirates; ^4^ Department of Biochemistry, College of Medicine and Health Science, United Arab Emirates University, Al Ain, United Arab Emirates; ^5^ Department of Pharmacology and Therapeutics, College of Medicine and Health Science, United Arab Emirates University, Al Ain, United Arab Emirates; ^6^ Ambulatory Healthcare Services, Al Maqtaa Healthcare Center, Middle Regions Clinics Division, SEHA, Abu Dhabi, United Arab Emirates; ^7^ Kings’ College London, Institute of Psychiatry, Psychology, Neuroscience, Department of Psychological Medicine, Centre for Affective Disorders, London, United Kingdom; ^8^ Behavioral Sciences Institute (BSI), Al Ain Hospital, SEHA, Al Ain, United Arab Emirates

**Keywords:** coronavirus, COVID-19, SARS-CoV-2, phenothiazine, antipsychotics, antiviral, chlorpromazine, clathrin

## Abstract

Coronavirus disease 2019 (COVID-19) is a severe acute respiratory syndrome (SARS) in humans that is caused by SARS-associated coronavirus type 2 (SARS-CoV-2). In the context of COVID-19, several aspects of the relations between psychiatry and the pandemic due to the coronavirus have been described. Some drugs used as antiviral medication have neuropsychiatric side effects, and conversely some psychotropic drugs have antiviral properties. Chlorpromazine (CPZ, Largactil^®^) is a well-established antipsychotic medication that has recently been proposed to have antiviral activity against SARS-CoV-2. This review aims to 1) inform health care professionals and scientists about the history of CPZ use in psychiatry and its potential anti- SARS-CoV-2 activities 2) inform psychiatrists about its potential anti-SARS-CoV-2 activities, and 3) propose a research protocol for investigating the use of CPZ in the treatment of COVID-19 during the potential second wave. The history of CPZ’s discovery and development is described in addition to the review of literature from published studies within the discipline of virology related to CPZ. The early stages of infection with coronavirus are critical events in the course of the viral cycle. In particular, viral entry is the first step in the interaction between the virus and the cell that can initiate, maintain, and spread the infection. The possible mechanism of action of CPZ is related to virus cell entry via clathrin-mediated endocytosis. Therefore, CPZ could be useful to treat COVID-19 patients provided that its efficacy is evaluated in adequate and well-conducted clinical trials. Interestingly, clinical trials of very good quality are in progress. However, more information is still needed about the appropriate dosage regimen. In short, CPZ repositioning is defined as a new use beyond the field of psychiatry.

## Introduction

The discovery of antidepressants has two parallel origins. On the one hand, the observation that an anti-tuberculosis drug, iproniazide chemically similar to isoniazid, improved the mood of *tuberculosis* patients and inhibited monoamine oxidase at the same time. On the other hand, a new molecule called imipramine was synthesized in order to obtain a neuroleptic like chlorpromazine, which had in fact not the expected but an antidepressant effect. Thus, two groups of antidepressants were born, MAOs and tricyclics. With the former, infectious disease medicine fueled the psychiatric practice. Could the opposite happen now? This article addresses this issue with the overall goals to: 1) educate health care professionals, including virologists, about the history of chlorpromazine (CPZ) use in psychiatry and its potential anti-SARS-CoV-2 activities, 2) inform psychiatrists about its potential anti-SARS-CoV-2 activities, and 3) propose a research protocol to investigate the use of CPZ in the treatment of COVID-19 for a potential second wave with psychiatric patients.

## Chlorpromazine and Coronavirus

The COVID-19 pandemic has had a major impact on mental health (Coccolini et al., 2020). The most common psychological and behavioral reactions are distress reactions which often include anxiety, insomnia, frustration, sense of insecurity, anger, increased use or avoidance of health services for fear of illness and indulging in risky maladaptive behaviors (for example, increased consumption of alcohol, illicit drugs and tobacco, change in work-life balance, social isolation, increased family conflicts and violent behavior) (Coccolini et al., 2020). Owing to these behaviors, psychiatrists have to be very involved, and at times, are obliged to adapt themselves due to the reorganization, redeployment and closing of services, following precautions for social distancing, use of telemedicine and wearing of personal protective equipment. They are not only repositioning themselves, but also offer repositioning of medication that they know very well. This is the case with the first known antipsychotic medication, CPZ (Plaze et al., 2020a; Plaze et al., 2020b; Stip, 2002; Stip, 2020). In 2020, a Canadian research letter (Stip, 2020) proposed the possibility that CPZ, a medication used in psychiatry for a long time, could potentially be used to counter COVID-19 (Nobile et al., 2020). The proposal was based on an old antimicrobial and antiviral CPZ data that had been documented when studying the history of the introduction of CPZ in North America (Stip, 2015). The first use in psychiatry of CPZ was in France and interestingly it is from there too that a concrete statement for CPZ repositioning for COVID-19 has just emerged (Plaze et al., 2020a; Plaze et al., 2020b). Since then, controlled clinical trials are underway with well-supported hypotheses and a rigorous methodology (NCT 04366739, Repurposing of Chlorpromazine in COVID-19 Treatment, reCoVery, France and NCT0434805, Egypt). CPZ is well-known to psychiatrists (Stip, 2000) and perhaps less well-known to virologists. In this article, we retrace the history of the use of CPZ in psychiatry and summarize the scientific arguments for prescribing CPZ in the context of viral infections, especially coronavirus infections due to the continuing COVID-19 pandemic.

Coronaviruses are a large group of non-segmented, (+) sense, enveloped RNA viruses with one of the largest genomes known, ranging from 27 to 33 kilo bases (kb). They cause a wide variety of diseases, ranging from respiratory, enteric, and hepatic to neurologic diseases (reviewed in Weiss and Leibowitz, 2011; Fehr and Perlman, 2015). Widely spread among the animal kingdom (mammals and birds), these viruses can be divided into four genera: α, β, γ, and δ. Coronaviruses are not new to the human species and so far, seven different strains of coronaviruses have crossed into the humans (HCoVs) belonging to either α or β coronavirus genera (Table 1; Ye et al., 2020). Of these, four strains cause mild upper respiratory infections of self-limiting nature, two of which were discovered in the 1960s (HCoV-OC43 and HCoV-229E). However, three other strains have entered the human species lately, causing a severe acute respiratory syndrome (SARS) in humans with high case fatality rates (CFR). The first such strain that had a CFR of 11% (Tsang et al., 2003), followed by Middle Eastern respiratory coronavirus (MERS-CoV) with a CFR of ∼35% (Bermingham et al., 2012; Khan and Sheek-Hussein, 2020), and the most recent one is SARS-CoV-2 in December 2019, the etiologic agent of COVID-19, with an evolving CFR of ∼2.4% (Wu F. et al., 2020; Wu A. et al., 2020; Coronaviridae Study Group of the International Committee on Taxonomy of Viruses, 2020; Uddin et al., 2020). Based on this species jump trajectory, further introductions of these viruses into humans are most likely; therefore, urgent efforts are needed to have effective therapies against these serious viral pathogens.

**TABLE 1:** Human coronaviruses (HCoVs) and their characteristics.

Human coronavirus	Genera	Entry receptor	Disease/Pathogenesis	References
HCoV-229E (1966)	α	Aminopeptidase (APN)	Upper respiratory tract infection with mild symptoms. Now endemic in human population, causing 15–30% of common colds	Hamre and Procknow (1966), Fehr and Perlman (2015)
HCoV-OC43 (1967)	β	Neu5, Ac2-containing moiety		McIntosh et al. (1967), Weiss and Navas-Martin (2005)
HCoV-NL63 (2004)	α	Angiotensin-converting enzyme 2 (ACE2)		van der Hoek et al. (2004), Fehr and Perlman (2015)
HCoV-HKU1 (2005)	β	?		Woo et al. (2005)
SARS-CoV-1 (2003)	β	ACE2	Other than symptoms of common cold and pneumonia, these viruses predominantly cause infection of lower respiratory tract causing acute respiratory distress syndrome (ARDS) and cytokine storm	Ksiazek et al. (2003), Drosten et al. (2003), Fehr and Perlman (2015)
MERS-CoV (2012)	β	Dipeptidyl peptidase 4 (DPP4)		Bermingham et al. (2012), Fehr and Perlman (2015)
SARS-CoV-2 (2019)	β	ACE2		Hoffman et al. (2020), Wu A. et al. (2020), Wu F. et al. (2020)

ARDS, acute respiratory distress syndrome; DPP4, dipeptidyl peptidase 4.

Among the three virulent strains of HCoVs, SARS-CoV-1 was the first human coronavirus that caused a global epidemic in 2003, spreading from Guangdong, China to over 12 dozen countries in several continents, causing 8,096 infections with 774 deaths (reviewed in Ye et al., 2020). It is thought to have originated in bats, followed by civet cats, and into humans. Other than fever, headaches, cough, and fatigue, patients infected with SARS-CoV-1 also displayed severe acute respiratory distress in the form of shortness of breath, atypical pneumonia, and cytokine storm. In 2012, MERS-CoV was identified (Zaki et al., 2012; Khan, 2013), again with origins in bats, but the dromedary camels were identified as the intermediate host for further transmission into humans (de Wit et al., 2016). Once in the human population, the virus spreads from person to person, primarily within the Middle East, but also leading to small outbreaks in Europe, Tunisia, and Korea (Ye et al., 2020). As of December 31, 2019, MERS-CoV had infected almost 2,500 individuals with 866 deaths, making it one of the most lethal human viruses known (Khan and Sheek-Hussein, 2020). Persons infected with MERS-CoV present with clinical symptoms resembling infection with SARS-CoV-1, except that some also show acute renal failure. Unlike SARS-CoV-1 and MERS-CoV, since its emergence in December 2019, SARS-CoV-2 has spread all over the world like wildfire, infecting >35 million people worldwide as of October 7, 2020 with over 1 million deaths across the world (COVID-19 Dashboard). Although SARS-CoV-2 is nearly 82% homologous to SARS-CoV-1 (Wu A. et al., 2020; Wu F. et al., 2020), SARS-CoV-2 is less lethal than SARS-CoV-1, but much more infectious. Other than symptoms of common cold and pneumonia, both viruses (SARS-CoV-1 and SARS-CoV-2) can cause acute respiratory distress syndrome, cytokine storm, and additionally diarrhea, unlike some of the other HCoVs (Ye et al., 2020). As can be seen from Table 1, the different HCoVs use different types of proteins as their receptors to enter cells. Interestingly, unlike MERS-CoV that uses the dipeptidyl peptidase 4 enzyme as its entry receptor, both SARS-CoV-1 and SARS-CoV-2 use the same receptor for entry into human cells, the angiotensin-converting enzyme 2 (ACE2) (Table 1), suggesting similar mechanism of entry into susceptible cells (Hoffman et al., 2020; Uddin et al., 2020).

Recently, Dyall and co-workers (Dyall et al., 2014) examined nearly 300 FDA approved drugs for antiviral activity against MERS-CoV and SARS-CoV-1 and found CPZ to be active against both of these coronaviruses. Similarly, de Wilde and co-workers found from a screening of 348 molecules that CPZ was one of the most promising agents for inhibiting coronaviruses in humans (de Wilde et al., 2014). Hence, CPZ merits further clinical investigation, in particular in a small-animal model for MERS-CoV infection. The fast worldwide spread of SARS-CoV-2 has created a need to find effective treatments. The repositioning of drugs already known and approved for a long time in humans is a practical and efficient approach to look for new therapeutic options in the face of this pandemic.

## Phenothiazines

CPZ belongs to the phenothiazine family of drugs that are primarily used for the treatment of schizophrenia and other forms of psychosis. Few psychiatrists know that methylene blue (one of the first antimalarial drugs) is a phenothiazine, with several biomedical and biological therapeutic facets (Henry et al., 2020). In addition to their antipsychotic activity, phenothiazines also have a significant antimicrobial effect, thanks to an action on the bactericidal function of macrophages and on inhibition efflux pumps (Chakrabarty et al., 1991; Barbe et al., 1995; Dastidar et al., 2013). They also eliminate bacterial resistance plasmids and destroy bacteria due to their membrane destabilizing effect. Methylene blue was one of the first synthetic drugs in medicine, with multiple indications, such as clinical pain syndromes, malaria, and psychotic disorders, and was used over a century ago (Bodoni, 1899; Stip, 2015). Methylene blue is a cationic thiazine dye with redox cycle properties and a selective affinity for the nervous system. Although CPZ was named as the first antipsychotic, methylene blue had actually been used to treat psychotic patients half a century earlier. In addition to treating psychotic patients, the use of methylene blue has also been explored in treating the bipolar disorder (Alda et al., 2017).

## Discovery of Chlorpromazine

CPZ is a neuroleptic,- a class of medication primarily used to manage psychosis. The first neuroleptic medication (Stip, 2002; Stip, 2015), CPZ was the product of research on antihistamines, discovered in 1937 by the Nobel Prize winner, Daniel Bovet. In 1944, he isolated phenothiazine and diethazine (Diparcol), which Jean Sigwald used in 1946 to treat Parkinson’s disease (Sigwald and Bouttier, 1953). Bernard Halpern had already introduced the use of phenothiazine antihistamines, such as promethazine (Phenergan), in medicine. In 1950, Paul Charpentier synthesized CPZ at the Rhône-Poulenc Laboratories (Stip, 2015). The drug was called 4560 RP and then Largactil in France. In 1952, Henri Laborit, Pierre Huguenard and Raymond Alluaume published the first use of 4560 RP (Stip, 2015). Afterwards, in his work on anesthesia, Laborit reported that some drowsiness was observed with 50–100 mg of intravenous CPZ, as well as, above all, a lack of interest of the patients to their surroundings. In December 1951, a clinical trial of 4560 RP was carried out by two psychiatrists, Jean Sigwald and Daniel Bouttier at the Paul Brousse Psychiatric Hospital in Paris: CPZ was effective in a series of cases of patients with hallucinations (Stip, 2015). Léon Chertok carried out what was probably the first clinical experience in a psychiatric environment with 4560 RP alone. It led to a normal subject experiment in Villejuif on a resident in psychiatry, Cornelia Quarti (Stip, 2015). In March 1952, three psychiatrists from the Val-de-Grâce Hospital in Paris, Hamon, Paraire and Velluz, published a case study of a patient suffering from manic attacks treated with CPZ, in association with a barbiturate, but the effect was insufficient (Hamon, 1952). Delay, Deniker, and Harl, published the first long-term observational study on May 26, 1952, on the occasion of the centenary of the Société Medico-Psychologique (Stip, 2015). The same team in Sainte-Anne Hospital in Paris published six articles over a period of 6 months, paving the way for the introduction of CPZ in psychiatry (Delay and Deniker, 1952; Delay et al., 1952). Initial trials outside of France, such as those in Padua with Rigotti, of Arnold in Vienna and Labhardt in Basel, Switzerland, produced similar results (Staehelin, 1954). The doses were then increased to 150–300 mg intramuscularly and 300–500 mg orally (Lemperiere and Ginestet, 2001). The first British report, by Anton-Stephens, identified indifference as the greatest effect on patients (Anton-Stephens, 1954). CPZ was introduced in 1954 in North America by two Canadian psychiatrists Roland Saucier and Heinz Lehmann (see Stip, 2015). By the end of 1955, there were also reports describing use of CPZ from Switzerland, Germany, Hungary, Latin America, Australia, Russia, and the United States (Bente and Itil, 1954; Sal et al., 1954; Staehelin, 1954; Kardos and Pertorini, 1955; Webb, 1955). CPZ was marketed in the United States as Thorazine by SmithKline, in France and England as Largactil by May and Baker (Stip, 2015), and in Japan by the name of Cotomin. The name of “Largactil” in French comes from its “broad action” because of the breadth (*largeur*) of its pharmacodynamic actions or due to its wide-ranging effects on different symptoms.

In 1957, the psychophysiological definition of neuroleptics was proposed based on five classical criteria: 1) creation of a state of psychomotor indifference, 2) reduction in states of excitement, agitation and aggressiveness, 3) progressive reduction of acute or chronic psychotic disorders, 4) neurological and neurovegetative side effects, and 5) predominantly subcortical in action. In the United States, the name of “neuroleptic class” was changed to “major tranquilizer” and “antipsychotic.” It took almost another 10 years to understand the mechanism of action of neuroleptics (antipsychotics), for the identification of dopaminergic receptors and the development of the dopamine (DA) hypothesis of schizophrenia with the contributions from Arvid Carlsson (Stip, 2002; Stip, 2015). This hypothesis received additional support with the correlation between clinical doses of CPZ and its power to block dopamine D2 (DA D2) receptors. Pharmacological interventions to treat psychosis have generally focused on modifying dysfunctional neurotransmitter systems to improve symptoms. So what can we learn from the discovery of CPZ? It is that a psychotropic drug can have the indication for a psychiatric disease before one knows its real mechanism of action.

## Chlorpromazine and Microbial Infections

Interestingly, Jean Delay’s book (Delay, 1950), published in 1950, 2 years before his articles on 4560 RP, indicates that antibiotics, such as penicillin, were among the recommended treatments for psychosis. With global use of CPZ, reports have shown that patients receiving CPZ had a lower incidence of bacterial infections (Kristiansen and Amaral, 1997). There is also growing evidence to suggest that inflammation, infection, oxidative stress, changes in the glutamatergic system, and neurotrophins are involved in schizophrenia (Hong and Bang, 2020). CPZ also has antimicrobial activity against *Staphylococcus aureus in vitro*, at concentrations greatly exceeding those achieved clinically (Ordway et al., 2002; Amaral and Molnar, 2012). The activity of CPZ against various microorganisms, including intracellular pathogens (leishmania, trypanosomes, amebae), has been studied both *in vitro* and *in vivo* (Molnar et al., 1976; Amaral and Molnar, 2012). These antimicrobial properties of CPZ reside in the side chains of the molecule and have led to the development of this phenothiazine as an anti-malarial agent (Molnar et al., 1976; Tsay, 2013). This creates a kind of loop, bringing us back to the malaria parasite, which was used to treat mental illness from the early 1920s to the late 1950s (Himmelweit, 1960; Tsay, 2013).

## Chlorpromazine and Viral Infections

Chlorpromazine has also shown antiviral activity against a number of viruses, including adeno (Diaconu et al., 2010), Ebola (Bhattacharyya et al., 2010), influenza (Rossman et al., 2012), and coronaviruses (De Wilde et al., 2014). Anti-viral activity of CPZ is mainly explained by inhibiting clathrin-mediated endocytosis (Wang et al., 1993; Joki-Korpela et al., 2001; Nawa et al., 2003; reviewed in Glebov, 2020; Yang and Shen, 2020). A key component of their virulence is the process of viral entry into host cells using the endocytic pathway, though other non-endosomal pathways may also be employed, depending upon the cell type and virus strain used (Inoue et al., 2007; Wang et al., 2008; reviewed in Glebov, 2020; Yang and Shen, 2020).

**FIGURE 1:**
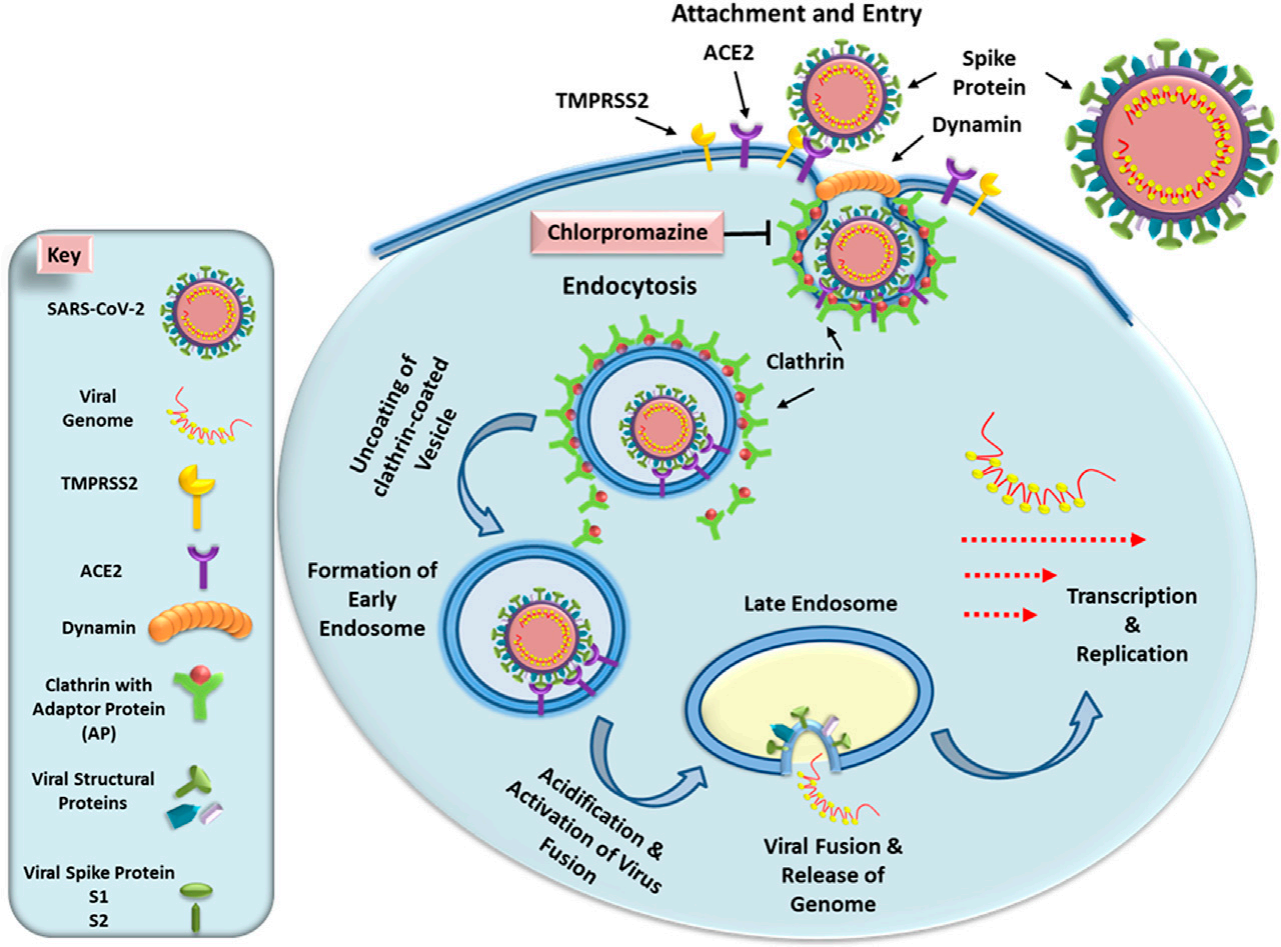
Schematic presentation of early steps of SARS-CoV-2 life cycle. The virus enters the cells by interacting with its receptor ACE2 protein using the spike protein and priming by the cell surface protease TMPRSS2. This induces formation of a vesicle via clathrin-dynamin-mediated endocytosis. The internalized vesicle loses its clathrin coat and fuses with an endosome. As the endosome undergoes acidification, it activates host proteases that induce fusion of the virus particle with the endosomal membrane, releasing the viral genomic RNA into the cytosol. Since SARS-CoV-2 RNA is (+) sense, it can immediately undergo translation and further transcription to allow the remaining steps of virus life cycle to continue. The left panel illustrates the key explaining the different components of this pathway. See text for details.

SARS-CoV-2 specifically enters its target cells by the binding of the viral spike protein S with the ACE2 cell surface receptor (Figure 1; Hoffman et al., 2020). Once attached to its target receptor, the virus is enclosed by the cell membrane which begins to form a vesicle (Burkard et al., 2014; Burkard et al., 2015). It rounds and stiffens due to the agglomeration of a cage of fibrous proteins, the clathrins, then separates from the cell membrane by gradually closing: it forms a neck which shrinks and disappears under the action of the dynamin, a protein that closes the bag. Then the vesicle loses its clathrins and fuses with an endosome. Endosome has a membrane not a wall and the fusion occurs between the viral envelope and the endosomal membrane. Under the action of a membrane protease, the virus fuses with the wall of the endosome (Figure 1). SARS-CoV-2 entry requires priming by cell-membrane bound proteases such as TMPRSS2. It is the S1 subunit that interacts with the ACE2 receptor via the receptor binding domain. The S2 domain initiates the process of membrane fusion via further activation by lysosomal proteases, allowing release of its genetic material within the target cell cytosol (Figure 1). This mode of penetration, known as “clathrin and dynamin-dependent,” is also observed with SARS-CoV-1, MERS-CoV, and also with the hepatitis C virus and the Influenza A virus (Zucker et al., 1990; Nawa et al., 2003; Inoue et al., 2007). Thus, infection of host cells by SARS-CoV-2 has been shown to be susceptible to lysosomotropic agents such as chloroquine that neutralize the acidic pH observed in the endosome-lysosomal compartments (Wang et al., 2008; Nobile et al., 2020). CPZ belongs to the family of cationic amphiphilic drugs and thus increase intra-vesicular pH of lysosomes. This blocks virus entry into cells by inhibiting activation of the lysosomal proteases that allow virus fusion with the endosomes and release of the viral genetic material into the cytosol. Based on these observations, the endocytic pathway comprising the endosome and the lysosome has become an important target for drug development in the fight against diseases caused by coronaviruses.

Thus, the early stages of viral infection are critical events in the course of the viral life cycle. In particular, viral entry is the first step in the interaction between a virus and a cell that can initiate, maintain and spread the infection (Joki-Korpela et al., 2001; Chu and Ng, 2004). Therefore, this stage constitutes a major target of the host’s adaptive immune response. The mouse hepatitis virus (MHV) has been used as a model for study coronavirus infections. In the case of a SARS-CoV-1 infection, similar to SARS-CoV-2, viral entry also requires a low pH in the intracytoplasmic vesicles; however, little is known about how SARS-CoV-1 invades these compartments (Subtil et al., 1995). Using the MHV model, experiments have revealed that viral entry mediated by clathrin-dependent endocytosis can be significantly inhibited by treatment with CPZ. Moreover, transfection of cells with small interfering RNAs specific to clathrin heavy chain can also inhibit viral gene expression, indicating the essential role of clathrin in viral entry (PuZhang, 2008). Similarly, entry of Zika virus has also been shown to be significantly inhibited by CPZ (Li et al., 2020). CPZ slows or suppresses the replication of alphaviruses, hepatitis C virus, SARS-CoV-1, and MHV-2. In fact, phenothiazines including CPZ, have various biological activities (Deetz et al., 2003). Phenothiazines have potent anti-plasmid and anti-bacterial activity *in vitro*. The benzo phenothiazine derivatives exhibit antibacterial activity *in vivo* and stimulate the differentiation of human myeloid leukemic cell lines (Motohashi et al., 2000; Huang et al., 2018). Thus, trifluoroperazine (Stelazine, Terfluzine) and CPZ have significant inhibitory effects on the replication of arenaviruses (Junin, Tacaribe and Pichinde viruses) (Candurra et al., 1996). *In vitro* antiviral activity on strains of herpes simplex virus type 2 (HSV-2) has also been demonstrated in cell cultures (Mitchell, 1994; Mucsi et al., 2001).

CPZ has also been shown to have affinity for sigma one receptors (Ki = 146 nM (Tam and Cook, 1984) and IC50 = 200 nM (Lang, 1995). Sigma one receptors are chaperone proteins found in the endoplasmic reticulum that may be responsible for proper folding of proteins after their translation. Being an enveloped virus, SARS-CoV-2 assembly takes place intracellularly where several of its structural proteins [spike (S), membrane (M), and envelope (E)] mature through the endoplasmic reticulum-golgi intermediate compartment (ERGIC) (Krijnse-Locker et al., 1994; Risco et al., 2002). This compartment is also used for the assembly of the virus particles that are transported to the cell surface in vesicles that are released via exocytosis. Thus, it is possible that CPZ may be acting not only at the entry phase of SARS-CoV-2 replication, but also at the later stages, potentially inhibiting the later stages of virus replication such as assembly and exocytosis from the infected cells. In fact, ERGIC has been proposed as a potential target for the investigation of antiviral drugs for SARS-CoV-2 (Tozzi, 2020).

In summary, CPZ as an anti-COVID-19 candidate is a fair illustration for repositioning the molecule as a treatment for COVID-19 with the findings of the two studies demonstrating *in vitro* activity against SARS-CoV-2 (Plaze et al., 2020b; Weston et al., 2020). These two pre-print studies demonstrate the *in vitro* activity of chlorpromazine against SARS-CoV-2 are crucial for proposing a repositioning.

Recently, French researchers (Plaze et al., 2020a; Plaze et al., 2020b; Nobile et al., 2020) have also identified several other mechanisms and advantages of chlorpromazine (Zucker et al., 1990; Tarazona et al., 1995). For example, CPZ stimulates the production of IgM. Moreover, its pulmonary concentration is 20–200 times higher than its plasma concentration. It is the same for the salivary concentration (20–60 times higher than that of plasma) and, of course, encephalic (25 times higher than that of plasma). This preferential distribution therefore seems particularly suitable for use in COVID-19 (Tarazona et al., 1995; Inoue et al., 2007).

## Dynamin Inhibition

Dynamin inhibition is an important part of the mechanism of action of CPZ (see Figure 1). This can be seen by the fact that there are many dynamin inhibitors available most of which are anti-infection in cells and a few in animal models, including bacterial, toxin and viral infections. The proof of dynamin being the mediator of this inhibition is commonly demonstrated by dominant-negative dynamin mutants (Harper et al., 2013).

Among the dynamin inhibitors, only the phenothiazines are clinically approved that are used at about the same concentrations and in clinically relevant levels. This potentially explains their mechanism of action and doses (Daniel et al., 2015). These concepts are important background that can actually be used to expand this hypothesis to other phenothiazines. Indeed, in March 2020, it was reported that the dynamin inhibitor of the phenothiazine class, prochlorperazine (prochlorperazine: Stemetil) blocks clathrin-mediated endocytosis in humans at clinical doses. This provides the evidence that the mechanism of action of phenothiazines is via inhibition of the clathrin-mediated endocytic pathway, thus strengthening the hypothesis (Chew et al., 2020). It is hard not to see that this extrapolates to all clinically-approved phenothiazines.

## Adverse Effects of Chlorpromazine

**FIGURE 2:**
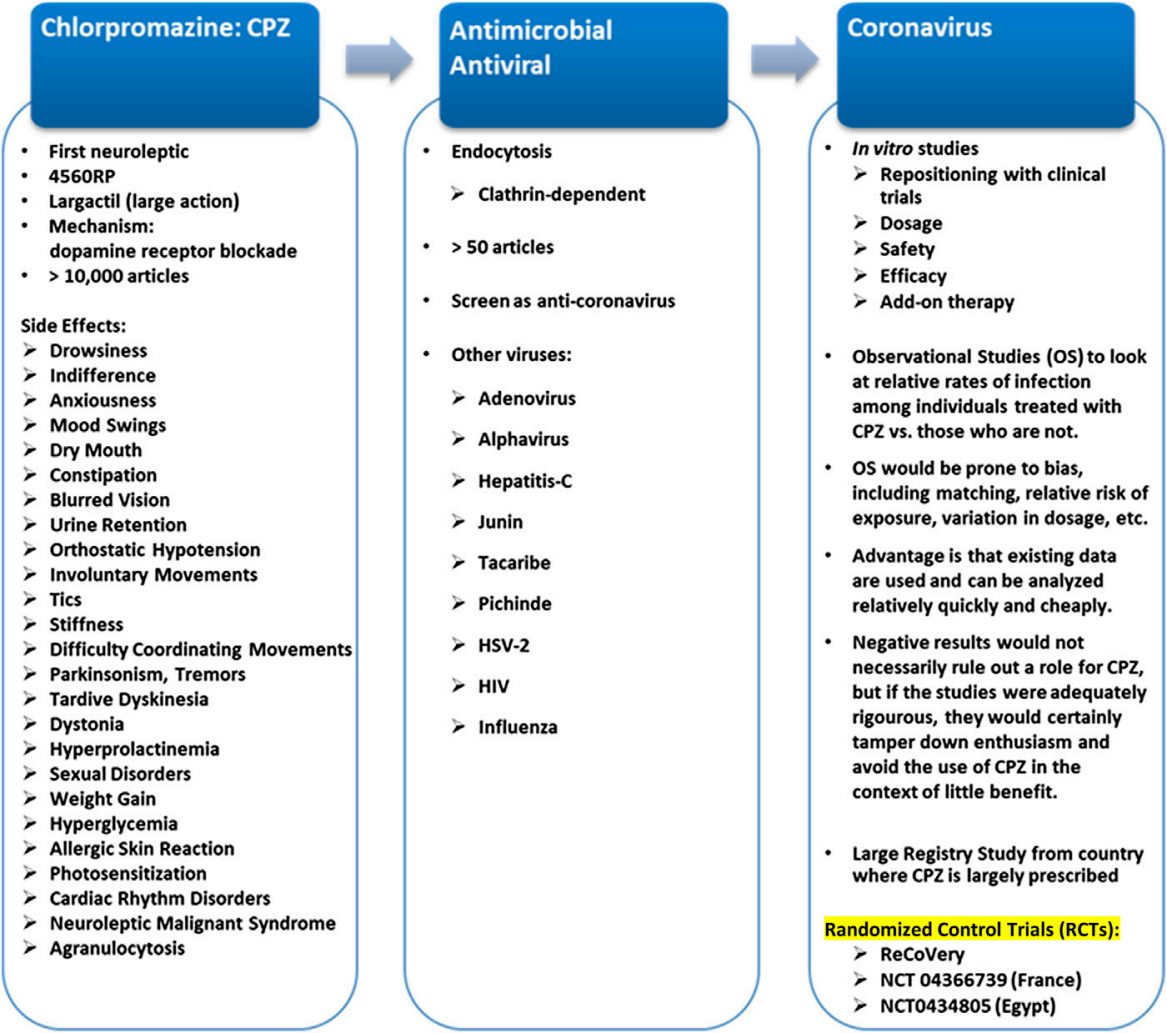
Repositioning of CPZ*. *Chlorpromazine in the world: Ampliactil (Argentina); Aspersinal (Argentina); Bellacina (Paraguay); Cepezet (Indonesia); Chlomazine (Japan); Chloractil (England); Chlorazin (Bulgaria, Switzerland); Chlormazine (Thailand); Chlorpromanyl (Canada); Chlorpromed (Thailand); Clonazine (Ireland); Clorpromaz (Brazil); Clozine (India); Contomin (Japan); Duncan (Thailand); Esmino (Japan); Fenactil (Poland); Hibernal (Hungary, Sweden); Klorproman (Czech Republic, Finland); Klorpromazin (Finland); Laractyl (Philippines); Largactil (Austria, Belgium, Canada, Chile, Costa Rica, Czech Republic, Denmark, Dominican Republic, Ecuador, El Salvador, England, Finland, France, Greece, Guatemala, Honduras, Hong Kong, Indonesia, Iran, Israel, Italy, Jordan, Mexico, Netherlands, Norway, Panama, Peru, Portugal, Puerto Rico, Spain, Switzerland, Uruguay, Venezuela); Largactil Forte (New Zealand); Matcine (China, Malaysia, Thailand); Megatil (India); Neomazine (Korea); Plegomazine (Iraq, Puerto Rico, Syria); Promactil (Indonesia); Promexin (Japan); Propaphenin (Germany); Prozil (Denmark); Prozin (Italy); Psynor (Philippines); Taroctyl (Israel); Thorazine (Philippines); Winsumin (Taiwan); Wintermin (Japan, Taiwan).

CPZ is widely used as an antipsychotic agent and is relatively safe to treat schizophrenia. Many side effects are linked to CPZ, including: drowsiness, indifference, anxious reaction, mood swings, dry mouth, constipation, blurred vision, urine retention, orthostatic hypotension, involuntary movements, tics, stiffness and difficulty coordinating movements, parkinsonism, tremor, tardive dyskinesia, dystonia, hyperprolactinemia, sexual side effects, weight gain, hyperglycemia, allergic skin reaction, photosensitization, QT prolongation and cardiac rhythm disorders, neuroleptic malignant syndrome and agranulocytosis (Figure 2; Solmi et al., 2017).

A recent meta-analysis from Huhn et al. (2019) compared the tolerability of 32 oral antipsychotics during the acute treatment of adults with multi-episode schizophrenia. Results for CPZ were given individually for six areas, namely: weight gain, extrapyramidal side effects, akathisia, raised prolactin, sedation, and anticholinergic side-effects compared with placebo. The results were then ranked cumulatively in comparison with all other treatments. In terms of weight gain, CPZ showed a mean difference (MD) of 2.37 (range = 1.43–3.32 kg), with a high level of confidence. This is the fourth highest out of all studied treatments (following zotepine, olanzapine, and sertindole), strongly favoring placebo treatment. The use of anti-Parkinson’s medication was used as a measure of extrapyramidal side-effects. CPZ showed a risk ratio (RR) of 2.17 (1.48–2.91) and was ranked 14th of the 33 treatments in favoring placebo treatment. For akathisia, CPZ showed a RR of 2.58 (1.30–4.30) and ranked 16 out of the 31 treatments in favoring placebo treatment. CPZ showed a MD in prolactin elevation of 8.70 (–8.16–25.75 ng/ml), ranking fifth out of the 21 treatments in favoring placebo treatment. For sedation, CPZ showed a RR of 2.55 (2.16–2.90). CPZ ranked sixth out of the 33 treatments in favoring placebo treatment. For anticholinergic side-effects, CPZ showed a RR of 2.58 (1.74–3.60), making it fifth out of the 32 antipsychotics in favoring placebo treatment.

New-generation neuroleptic agents were found to cause fewer unwanted extrapyramidal side effects than the traditional antipsychotic drugs (Solmi et al., 2017). However, they are not phenothiazines.

Overall, the tolerability data for CPZ favored placebo treatment, with six side effects all favoring placebo treatment. For all-cause discontinuation, CPZ showed a RR of 0.91, ranking eighth out of the 33 treatments in favoring placebo treatment. However, the quality of the data was rated as either low or very low for several of the side effects; only the data for weight gain was ranked as high quality and the data for sedation was ranked as moderate quality. Furthermore, there was no usable data for CPZ on corrected QT prolongation. This suggests that there is an urgent need for more quality randomised controlled trials.

## Chlorpromazine as an Anti-COVID-19 Candidate

Because CPZ has been used for over 70 years, many pharmacological and safety data are easily available. Moreover, studies of the biological effects of CPZ have generated almost 20,000 published studies listed in PubMed, second only to Aspirin (over 50,000), largely owing to its abundant side effects. In Tokyo, November 1962, in a symposium on Japanese Encephalitis and other arboviral infections, the WHO reported: “*In the acute stage of the disease, the use of tepid sponging, ice packs, oxygen therapy, corticosteroids, salicylates, chlorpromazine may be effective*” [World Health Organization. Regional Office for the Western Pacific & Seminar on Japanese Encephalitis and other Arbovirus Infections (World Health Organization, 1962)]*.* To our knowledge, there is no epidemiological or observational study exploring the clinical status of COVID-19 patients under CPZ treatment. When faced with this alarming pandemic and given the concerns about the potentially high mortality rate, clinicians and patients will be tempted to try unproven therapies. CPZ repositioning includes a new use outside of psychiatry. The mechanism of action of CPZ is either via inhibition of clathrin-mediated endocytosis, and/or at later stages of virus assembly and egress which is not well known to psychiatrists. Conversely, virologists do not know very well its psychotropic effect. Thus, CPZ could prove efficacious in treating COVID-19 patients, provided adequate and good clinical trials are conducted and the results analyzed.

## Proposal for a Research Protocol: PsyCHovid

CPZ, in the context of the pandemic, could be helpful for the vulnerable psychiatric population. Psychotic patients are suffering much more often than the general population from comorbidities (cardiovascular pathologies, diabetes, obesity, smoking) which are risk factors for severe SARS-CoV-2 infection (Fonseca et al., 2020). Usually, without the pandemic context, patients hospitalized in psychiatry suffering from psychosis present a high-risk of pneumococcal infections (Seminog and Goldacre, 2013). In this context, we present a research protocol which can be conducted in a multisite setting for patients treated for psychosis and who develop COVID-19 (see Supplementary Material). The goal of this randomized controlled clinical trial would be to assess if CPZ is beneficial to limit the symptoms, and consequences of COVID-19 and maintain or improve psychotic symptoms as an add-on medication administered early at the onset of symptoms to the usual and adjusted antipsychotic treatment. It should be a randomized control trial with a comparison to treatment as usual arm without add-on CPZ (see Supplementary Material). Patients in the experimental arm should receive CPZ and should maintain their current treatment with an antipsychotic medication. To avoid increasing side effects due to antipsychotic medication, a down titration of their current treatment should be prescribed. The adjustment of the decreased dose of the antipsychotic patients for those who are in the experimental arm should be based on the well-known CPZ–Equivalent (CPZeq). The concept of CPZeq was derived from the potency for dopamine receptor blockade, which was determined empirically by judging the dose equivalence between different antipsychotic agents. The dosage of the add-on CPZ should be between 50 and 100 mg at night, according to the tolerability and the stability of the medical and psychiatric conditions. The outcome should be the efficacy of the symptoms related to COVID-19 and the global tolerability to CPZ (see Supplementary Material).

At this stage, the clinical trial framework we propose is a little simplistic in design, and it is not compared to design of the existing reCoVery trial (ClinicalTrials.gov Identifier: NCT04366739) for any new or unique elements. It does not include other phenothiazines, and is limited to psychiatric patients.

In fact, it could be an issue that this COVID-19 potential therapy would be limited to only psychiatric patients due to the drowsinees, and other features of CPZ. But why not if we consider psychotic patients very vulnerable and very often left out? While not ignored here, we leave a place for discussion to explore which patient population could be impacted or benefit by a successful trial outcome. CPZ is a better candidate against SARS-CoV-2 than some other medications. This property is its easy penetration in the brain. Indeed, more and more studies are emerging on the neurological complications (encephalitis, cognitive impairments, psychiatric disease such as depression, psychosis…) provoked by SARS-CoV-2 since it passes the blood brain barrier. It is thus important to find molecules that also pass the blood brain barrier in order to prevent neurological damages. The publication (Chew et al., 2020) indeed points to prochlorperazine as a potential better candidate for this proposed trial than CPZ, due to far less CNS penetration and reduced drowsiness (better side-effect profile).

Furthermore, it would be interesting to investigate the combined benefit of antipsychotic and antiviral activity of CPZ in patients with COVID-19 and psychosis. People with preexisting mental disorders may become more vulnerable during an epidemic (Chevance et al., 2020; Vigo et al., 2020) and any strategy that can simplify their lives and the appropriate follow-up will be welcome.

## Conclusion

CPZ is one of the most widely used treatments for schizophrenia worldwide and remains low-cost and widely available. Despite its known side effects, CPZ is likely to remain a benchmark drug. This article makes a case for testing CPZ as a possible treatment for the COVID-19 infection. To engage the interest of virologists about a psychiatric drug, we presented in this review much historical information that provides a narrative perspective on what is otherwise a straightforward and rather “dry” reminder that some psychiatric medications may have useful antiviral properties. However, in the end, there is only scarce evidence that this drug will work, so this becomes a call to action for future studies. Currently, there are some limited, but encouraging clinical implications for these ideas with good quality controlled trials registered and in progress in France and Egypt (NCT 04366739, NCT0434805). The overview of the evidence, mainly *in vitro*, is informative. The various ways in which CPZ might have antiviral properties relative to COVID-19 might be helpful. In the end, the conclusion suggests that CPZ should probably be tried.

## Author Contributions

ES designed and directed the project, developed the theory and performed the review of literature, referred to his previous work on Chlorpromazine, and finalized the article. All other authors contributed to the article by performing review of the literature in their respective areas, writing and improving different portions of the article, and approved the submitted version.

## Conflict of Interest

The authors declare that the research was conducted in the absence of any commercial or financial relationships that could be construed as a potential conflict of interest.

## References

[B1] Alda M. McKinnon M. Blagdon R. Garnham J. MacLellan S. O’Donovan C. (2017). Methylene blue treatment for residual symptoms of bipolar disorder: randomised crossover study. Br. J. Psychiatr. 210 (1), 54–60. 10.1192/bjp.bp.115.173930 27284082

[B2] Amaral L. Molnar J. (2012). Why and how the old neuroleptic thioridazine cures the XDR-TB patient. Pharmaceuticals (Basel) 5 (9), 1021–1031. 10.3390/ph5091021 24280703 PMC3816647

[B3] Anton-Stephens D. (1954). Preliminary observations on the psychiatric uses of chlorpromazine (Largactil). J. Ment. Sci. 100, 543–557. 10.1192/bjp.100.419.543 13175016

[B4] Barbe J. Keyzer H. Soyfer J. (Editors) (1995). Biological and chemical aspects of thiazines and analogs. San Gabriel, CA: Enlight Associates, 347–356.

[B5] Bente D. Itil T. M. (1954). Zur wirkung des phenothiazin körpers megaphen auf das menschliche hirnströmbild. Arzneim. Forsch. 4, 418–423. 13198728

[B6] Bermingham A. Chand M. A. Brown C. S. Aarons E. Tong C. Langrish C. (2012). Severe respiratory illness caused by a novel coronavirus, in a patient transferred to the United Kingdom from the Middle East, September 2012. Euro Surveill. 17, 20290. 23078800

[B7] Bhattacharyya S. Warfield K. L. Ruthel G. Bavari S. Aman M. J. Hope T. J. (2010). Ebola virus uses clathrin-mediated endocytosis as an entry pathway. Virology 401 (1), 18–28. 10.1016/j.virol.2010.02.015 20202662 PMC3732189

[B8] Bodoni M. (1899). La bleu de methylene comme calmant chez les alienes. Sem. Med. 7, 56

[B9] Burkard C. Verheije M. H. Haagmans B. L. van Kuppeveld F. J. Rottier P. J. Bosch B. J. (2015). ATP1A1-mediated Src signaling inhibits coronavirus entry into host cells. J. Virol. 89 (8), 4434–4448. 10.1128/JVI.03274-14 25653449 PMC4442369

[B10] Burkard C. Verheije M. H. Wicht O. van Kasteren S. I. van Kuppeveld F. J. Haagmans B. L. (2014). Coronavirus cell entry occurs through the endo-/lysosomal pathway in a proteolysis-dependent manner. PLoS Pathogens 10 (11), e1004502 10.1371/journal.ppat.1004502 25375324 PMC4223067

[B11] Candurra N. A. Maskin L. Damonte E. B. (1996). Inhibition of arenavirus multiplication *in vitro* by phenothiazines. Antivir. Res. 31 (3), 149–158. 10.1016/0166-3542(96)06956-2 8811199

[B12] Chakrabarty a. N. sen t. banerji S. (1991). Effects of structural modifications on autimicrobial actious of promethazine, methdilazine and related phenothiazines. Thiazines Related Compounds 22, 219–233.

[B13] Chan-Yeung M. Xu R. H. (2003). *SARS: epidemiology* . Respirology 8 (Suppl. l), S9–S14. 10.1046/j.1440-1843.2003.00518.x 15018127 PMC7169193

[B14] Chen Y. Liu Q. Guo D. (2020). Emerging coronaviruses: genome structure, replication, and pathogenesis. J. Med. Virol. 92 (4), 418–423. 10.1002/jmv.25681 31967327 PMC7167049

[B15] Chevance A. Gourion D. Hoertel N. Llorca P.-M. Thomas P. Bocher R. (2020). [Ensuring mental health care during the SARS-CoV-2 epidemic in france: a narrative review]. Encephale 46 (3S), S3–S13. 10.1016/j.encep.2020.03.001 32370982 PMC7174154

[B16] Chew H. Y. De Lima P. O. Gonzalez Cruz J. L. Banushi B. Echejoh G. Hu L. (2020) Endocytosis inhibition in humans to improve responses to ADCC-mediating antibodies. Cell 180, 895–914.e27. 10.1016/j.cell.2020.02.019 32142680

[B17] Chu J. J. Ng M. L. (2004). Infectious entry of West Nile virus occurs through a clathrin-mediated endocytic pathway. J. Virol. 78 (19), 10543–10555. 10.1128/JVI.78.19.10543-10555.2004 15367621 PMC516396

[B18] Coccolini F. Sartelli M. Kluger Y. Pikoulis E. Karamagioli E. Moore E. E. (2020). COVID-19 the showdown for mass casualty preparedness and management: the cassandra syndrome. World J. Emerg. Surg. 15 (1), 26 10.1186/s13017-020-00304-5 32272957 PMC7145275

[B19] Coronaviridae Study Group of the International Committee on Taxonomy of Viruses (2020). The species Severe acute respiratory syndrome-related coronavirus: classifying 2019-nCoV and naming it SARS-CoV-2. Nat. Microbiol. 5, 536–544. 10.1038/s41564-020-0695-z 32123347 PMC7095448

[B20] CSSE at Johns Hopkins University (JHU) (2020). COVID-19 dashboard by center for systems science and engineering. Available at: https://coronavirus.jhu.edu/map.html (Accessed June 27, 2020).

[B21] Daniel J. A. Chau N. Abdel-Hamid M. K. Hu L. von Kleist L. Whiting A. (2015) Phenothiazine-derived antipsychotic drugs inhibit dynamin and clathrin-mediated endocytosis. Traffic 15, 635–654. 10.1111/tra.12272 25693808

[B22] Dastidar S. G. Kristiansen J. E. Molnar J. Amaral L. (2013). Role of phenothiazines and structurally similar compounds of plant origin in the fight against infections by drug resistant bacteria. Antibiotics 2 (1), 58–72. 10.3390/antibiotics2010058 27029292 PMC4790298

[B23] De Wilde A. H. Jochmans D. Posthuma C. C. Zevenhoven-Dobbe J. C. Van Nieuwkoop S. Bestebroer T. M. (2014). Screening of an FDA-approved compound library identifies four small-molecule inhibitors of Middle East respiratory syndrome coronavirus replication in cell culture. Antimicrob. Agents Chemother. 58 (8), 4875–4884. 10.1128/AAC.03011-14 24841269 PMC4136071

[B24] de Wit E. van Doremalen N. Falzarano D. Munster V. J. (2016). SARS and MERS: recent insights into emerging coronaviruses. Nat. Rev. Microbiol. 14 (8), 523–534. 10.1002/jgm.1453 27344959 PMC7097822

[B25] Deetz T. R. Sawyer M. H. Billman G. Schuster F. L. Visvesvara G. S. (2003). Successful treatment of Balamuthia amoebic encephalitis: presentation of 2 cases. Clin. Infect. Dis. 37 (10), 1304–1312. 10.1086/379020 14583863

[B26] Delay J. (1950). “Nouvelles chimiothérapies en psychiatrie,” in Méthodes biologiques en clinique psychiatrique (Paris, FR: Masson), 375–382.

[B27] Delay J. Deniker P. (1952). 38 cas de psychoses traitèes par la cure prolongèe et continuè de 4560 RP. CR Congr. Méd. Alién Neurol. (France) 50, 503–513

[B28] Delay J. Deniker P. Harl J. M. (1952). [Therapeutic use in psychiatry of phenothiazine of central elective action (4560 RP)]. Ann. Med. Psychol. (Paris) 110, 112–117 12986408

[B29] Diaconu I. Cerullo V. Escutenaire S. Kanerva A. Bauerschmitz G. J. Hernandez-Alcoceba R. (2010). Human adenovirus replication in immunocompetent Syrian hamsters can be attenuated with chlorpromazine or cidofovir. J. Gene Med. 12 (5), 435–445. 10.1002/jgm.1453 20440754

[B30] Drosten C. Günther S. Preiser W. van der Werf S. Brodt H. R. Becker S. (2003). Identification of a novel coronavirus in patients with severe acute respiratory syndrome. N. Engl. J. Med. 348 (20), 1967–1976. 10.1056/NEJMoa030747 12690091

[B31] Dyall J. Coleman C. M. Hart B. J. Venkataraman T. Holbrook M. R. Kindrachuk J. (2014). Repurposing of clinically developed drugs for treatment of Middle East respiratory syndrome coronavirus infection. Antimicrob. Agents Chemother. 58 (8), 4885–4893. 10.1128/AAC.03036-14 24841273 PMC4136000

[B32] Fehr A. R. Perlman S. (2015). Coronaviruses: an overview of their replication and pathogenesis. Methods Mol. Biol. 1282, 1–23. 10.1007/978-1-4939-2438-7_1 25720466 PMC4369385

[B33] Fonseca L. Diniz E. Mendonça G. Malinowski F. Mari J. Gadelha A. (2020). Schizophrenia and COVID-19: risks and recommendations. Braz. J. Psychiatry 42 (3), 236–238. 10.1590/1516-4446-2020-0010 32294689 PMC7236151

[B34] Glebov O. O. (2020). Understanding SARS-CoV-2 endocytosis for COVID-19 drug repurposing. FEBS J. [Epub ahead of print]. 10.1111/febs.15369 PMC7276759 32428379

[B35] Hamon J. (1952). Remaeques sur l’action du 4560 RP sur l’agitation maniaque. Ann. Med. Psychol. 110, 331–335. 12976825

[B36] Hamre D. Procknow J. J. (1966). A new virus isolated from the human respiratory tract. Proc. Soc. Exp. Biol. Med. 121, 190–193. 10.3181/00379727-121-30734 4285768

[B37] Harper C. B. Popoff M. R. McCluskey A. Robinson P. J. Meunier F. A. (2013) Targeting membrane trafficking in infection prophylaxis: dynamin inhibitors. Trends Cell Biol. 23, 90–101. 10.1016/j.tcb.2012.10.007 23164733

[B38] Hayashi T. Su T.-P. (2004). Sigma-1 receptor ligands: potential in the treatment of neuropsychiatric disorders. CNS Drugs 18, 269–284. 10.2165/00023210-200418050-00001 15089113

[B39] Henry M. Summa M. Patrick L. Schwartz L. (2020). A cohort of cancer patients with no reported cases of SARS-CoV-2 infection: the possible preventive role of Methylene Blue. Substantia, 888.

[B40] Himmelweit F. (Editor) (1960). The collected papers of Paul Ehrlich. 1st Edition Pergamon Press.

[B41] Hoffmann M. Kleine-Weber H. Schroeder S. Krüger N. Herrler T. Erichsen S. (2020). SARS-CoV-2 cell entry depends on ACE2 and TMPRSS2 and is blocked by a clinically proven protease inhibitor. Cell 181 (2), 271–280.e8. 10.1016/j.cell.2020.02.052 32142651 PMC7102627

[B42] Hong J. Bang M. (2020). Anti-inflammatory strategies for schizophrenia: a review of evidence for therapeutic applications and drug repurposing. Clin. Psychopharmacol. Neurosci. 18 (1), 10–24. 10.9758/cpn.2020.18.1.10 31958901 PMC7006977

[B43] Huang J. Zhao D. Liu Z. Liu F. (2018). Repurposing psychiatric drugs as anti-cancer agents. Cancer Lett. 419, 257–265. 10.1016/j.canlet.2018.01.058 29414306

[B44] Huhn M. Nikolakopoulou A. Schneider-Thoma J. Krause M. Samara M. Peter N. (2019). Comparative efficacy and tolerability of 32 oral antipsychotics for the acute treatment of adults with multi-episode schizophrenia: a systematic review and network meta-analysis. Lancet 394 (10202), 939–951. 10.1016/S0140-6736(19)31135-3 31303314 PMC6891890

[B45] Inoue Y. Tanaka N. Tanaka Y. Inoue S. Morita K. Zhuang M. (2007). Clathrin-dependent entry of severe acute respiratory syndrome coronavirus into target cells expressing ACE2 with the cytoplasmic tail deleted. J. Virol. 81 (16), 8722–8729. 10.1128/jvi.00253-07 17522231 PMC1951348

[B46] Joki-Korpela P. Marjomäki V. Krogerus C. Heino J. Hyypiä T. (2001). Entry of human parechovirus 1. J. Virol. 75 (4), 1958–1967. 10.1128/jvi.75.4.1958-1967.2001 11160695 PMC115142

[B47] Kardos G. Pertorini R. (1955). Largactil a psychiátriában. Ideggyogyaszati Szle. 8, 65–61. 13345375

[B48] Khan G. (2013). A novel coronavirus capable of lethal human infections: an emerging picture. Virol. J. 10, 66 10.1186/1743-422X-10-66 23445530 PMC3599982

[B49] Khan G. Sheek-Hussein M. (2020). The Middle East respiratory syndrome coronavirus: an emerging virus of global threat. Emerging Reemerging Viral Pathog., 151–167. 10.1016/B978-0-12-819400-3.00008-9

[B50] Krijnse-Locker J. Ericsson M. Rottier P. J. Griffiths G. (1994). Characterization of the budding compartment of mouse hepatitis virus: evidence that transport from the RER to the Golgi complex requires only one vesicular transport step. J. Cell Biol. 124 (1-2), 55–70. 10.1083/jcb.124.1.55 8294506 PMC2119890

[B51] Kristiansen J. E. Amaral L. (1997). The potential management of resistant infections with non-antibiotics. J. Antimicrob. Chemother. 40 (3). 319–327. 10.1093/jac/40.3.319 9338482

[B52] Ksiazek T. G. Erdman D. Goldsmith C. S. Zaki S. R. Peret T. Emery S. (2003). SARS Working Group. A novel coronavirus associated with severe acute respiratory syndrome. N. Engl. J. Med. 348 (20), 1953–1966. 10.1056/NEJMoa030781 12690092

[B53] Lang A. (1995). The role of dopamine, 5-hydroxytryptamine, sigma and NMDA receptors in the action of antipsychotic drugs. Tartu: Tartu University Press

[B54] Lemperiere T. Ginestet D. (2001). “Les medicaments antipsychotiques. Début et étapes de la découverte des neuroleptiques”, in Médicaments antipsychotiques. Editors Olié J. P. Dalery J. Azorin J. M. (Paris, FR: Acanthe), 3–22.

[B55] Li M. Zhang D. Li C. Zheng Z. Fu M. Ni F. (2020). Characterization of Zika virus endocytic pathways in human glioblastoma cells. Front. Microbiol. 11, 242 10.3389/fmicb.2020.00242 32210929 PMC7069030

[B56] Lu R. Zhao X. Li J. Niu P. Yang B. Wu H. (2020). Genomic characterisation and epidemiology of 2019 novel coronavirus: implications for virus origins and receptor binding. Lancet 395 (10224), 565–574. 10.1016/S0140-6736(20)30251-8 32007145 PMC7159086

[B57] McIntosh K. Dees J. H. Becker W. B. Kapikian A. Z. Chanock R. M. (1967). Recovery in tracheal organ cultures of novel viruses from patients with respiratory disease. Proc. Natl. Acad. Sci. U.S.A. 57 (4), 933–940. 10.1073/pnas.57.4.933 5231356 PMC224637

[B58] Mitchell S. C. (1994). The toxicity of phenothiazine. Drug Metabol. Drug Interact. 11 (3), 201–236. 10.1515/DMDI.1994.11.3.201 12371440

[B59] Molnar J. Mandi Y. Kiraly J. (1976). Antibacterial effect of some phenothiazine compounds and B factor elimination by chloropromazine. Acta Microbiol. Acad. Sci. Hungar. 23 (1), 45–54. 10.1128/aem.19.6.1017-1018.1970 820163

[B60] Motohashi N. Kawase M. Saito S. Sakagami H. (2000). Antitumor potential and possible targets of phenothiazine-related compounds. Curr. Drug Targets 1 (3), 237–246. 10.2174/1389450003349191 11465073

[B61] Mucsi I. Molnár J. Motohashi N. (2001). Combination of benzo [a] phenothiazines with acyclovir against herpes simplex virus. Int. J. Antimicrob. Agents 18 (1), 67–72. 10.1016/s0924-8579(01)00323-5 11463529

[B62] Nawa M. Takasaki T. Yamada K. I. Kurane I. Akatsuka T. (2003). Interference in Japanese encephalitis virus infection of Vero cells by a cationic amphiphilic drug, chlorpromazine. J. Gen. Virol. 84 (7), 1737–1741. 10.1099/vir.0.18883-0 12810867

[B63] Nobile B. Durand M. Courtet P. Van de Perre P. Nagot N. Molès J. P. (2020). Could the antipsychotic chlorpromazine be a potential treatment for SARS-CoV-2? Schizophr. Res. [Epub ahead of print]. 10.1016/j.schres.2020.07.015 PMC7381925 32773341

[B64] Ordway D. Viveiros M. Leandro C. Arroz M. J. Molnar J. Kristiansen J. E. (2002). Chlorpromazine has intracellular killing activity against phagocytosed *Staphylococcus aureus* at clinical concentrations. J. Infect. Chemother. 8 (3), 227–231. 10.1007/s10156-002-0188-4 12373485

[B65] Plaze M. Attali D. Petit A.-C. Blatzer M. Simon-Loriere E. Vinckier F. (2020a). [Repurposing of chlorpromazine in COVID-19 treatment: the reCoVery study]. L’Encephale 46 (3), 169–172. 10.1016/j.encep.2020.04.010 PMC7229964 32425222

[B66] Plaze M. Attali D. Prot M. Petit A. C. Blatzer M. Vinckier F. (2020b). Inhibition of the replication of SARS-CoV-2 in human cells by the FDA-approved drug chlorpromazine. bioRxiv. 10.1016/j.ijantimicag.2020.106274 PMC7772996 33387629

[B67] Pu Y. Zhang X. (2008). Mouse hepatitis virus type 2 enters cells through a clathrin-mediated endocytic pathway independent of Eps15. J. Virol. 82 (16), 8112–8123. 10.1128/JVI.00837-08 18550663 PMC2519582

[B68] Risco C. Rodríguez J. R. López-Iglesias C. Carrascosa J. L. Esteban M. Rodríguez D. (2002). Endoplasmic reticulum-Golgi intermediate compartment membranes and vimentin filaments participate in vaccinia virus assembly. J. Virol. 76 (4), 1839–1855. 10.1128/jvi.76.4.1839-1855.2002 11799179 PMC135913

[B69] Rossman J. S. Leser G. P. Lamb R. A. (2012). Filamentous influenza virus enters cells via macropinocytosis. J. Virol. 86 (20) 10950–10960. 10.1128/JVI.05992-11 22875971 PMC3457176

[B70] Sal Y. Rosas F. Jerí R. Sanchez J. (1954). Chlorpromazine in neuropsychiatry. J. Am. Med. Assoc. 156, 558.

[B71] Seminog O. O. Goldacre M. J. (2013). Risk of pneumonia and pneumococcal disease in people with severe mental illness: english record linkage studies. Thorax 68 (2), 171–176. 10.1136/thoraxjnl-2012-202480 23242947

[B72] Sigwald J. Bouttier D. (1953). Le chlorhydrate de de chloro-3 (dimethylamino-3'-propyl)-10-phénothiazine en pratique neuro-psychiatrique courante. Ann. Med. Interne. 54 (2), 150–182. 13065806

[B73] Solmi M. Murru A. Pacchiarotti I. Undurraga J. Veronese N. Fornaro M. (2017). Safety, tolerability, and risks associated with first-and second-generation antipsychotics: a state-of-the-art clinical review. Therapeut. Clin. Risk Manag. 13, 757 10.2147/TCRM.S117321 PMC5499790 28721057

[B74] Staehelin J. E. (1954). Einige allgemeine bemerkungen uber die largactiltherapie in der psychiatrischen universitatsklinik basel. Schweiz. Arch. Neurol. Psychiatr. 73, 288–291. 13195582

[B75] Stip E. (2000). Novel antipsychotics: issues and controversies. typicality of atypical antipsychotics. J. Psychiatry Neurosci. 25 (2), 137–153. 10740987 PMC1408068

[B76] Stip E. (2002). Happy birthday neuroleptics! 50 years later: la folie du doute. Eur. Psychiatry 17 (3), 115–119. 10.1016/S0924-9338(02)00639-9 12052571

[B77] Stip E. (2015). Who pioneered the use of antipsychotics in north america? Can. J. Psychiatry 60 (3), S5–S13. PMC4418623 25886681

[B78] Stip E. (2020). Psychiatry and COVID-19: the role of chlorpromazine. Can. J. Psychiatry 65 (10), 739–740. 10.1177/0706743720934997 32536208 PMC7502873

[B79] Subtil A. Hemar A. Dautry‐Varsat A. (1995). Rapid endocytosis of interleukin 2 receptors when clathrin‐coated pit endocytosis is inhibited. Biol. Cell. 84 (1–2), 96 10.1016/0248-4900(96)81369-4 7706397

[B80] Tam S. W. Cook L. (1984). Sigma opiates and certain antipsychotic drugs mutually inhibit (+)-[3H] SKF 10,047 and [3H]haloperidol binding in Guinea pig brain membranes. Proc. Natl. Acad. Sci. U.S.A. 81 (17), 5618–5621. 10.1073/pnas.81.17.5618 6147851 PMC391758

[B81] Tarazona R. González-García A. Zamzami N. Marchetti P. Frechin N. Gonzalo J. A. (1995). Chlorpromazine amplifies macrophage-dependent IL-10 production *in vivo* . J. Immunol. 154 (2), 861–870 7814889

[B82] Tozzi A. (2020). Rapid Response: endoplasmic reticulum-golgi intermediate compartment: a novel target for SARS-COV-2 therapies? BMJ 368, m1252 10.1136/bmj.m1252 32217607

[B83] Tsang K. W. Ho P. L. Ooi G. C. Yee W. K. Wang T. Chan-Yeung M. (2003). A cluster of cases of severe acute respiratory syndrome in hong kong. N. Engl. J. Med. 348 (20), 1977–1985. 10.1056/NEJMoa030666 12671062

[B84] Tsay C. J. (2013). Julius Wagner-Jauregg and the legacy of malarial therapy for the treatment of general paresis of the insane. Yale J. Biol. Med. 86 (2), 245–254 23766744 PMC3670443

[B85] Uddin M. Mustafa F. Rizvi T. A. Loney T. Suwaidi H. A. Al-Marzouqi A. (2020). SARS-CoV-2/COVID-19: viral genomics, epidemiology, vaccines, and therapeutic interventions. Viruses 12 (5), 526 10.3390/v12050526 PMC7290442 32397688

[B86] van der Hoek L. Pyrc K. Jebbink M. F. Vermeulen-Oost W. Berkhout R. J. Wolthers K. C. (2004). Identification of a new human coronavirus. Nat. Med. 10 (4), 368–373. 10.1038/nm1024 15034574 PMC7095789

[B87] Vigo D. Patten S. Pajer K. Krausz M. Taylor S. Rush B. (2020). Mental health of communities during the COVID-19 pandemic. Can. J. Psychiatr. 65 (10), 681–687. 10.1177/0706743720926676 PMC7502878 32391720

[B88] Wang H. Yang P. Liu K. Guo F. Zhang Y. Zhang G. (2008). SARS coronavirus entry into host cells through a novel clathrin- and caveolae-independent endocytic pathway. Cell Res. 18 (2), 290–301. 10.1038/cr.2008.15 18227861 PMC7091891

[B89] Wang L. H. Rothberg K. G. Anderson R. G. W. (1993). Mis-assembly of clathrin lattices on endosomes reveals a regulatory switch for coated pit formation. J. Cell Biol. 123 (5), 1107–1117. 10.1083/jcb.123.5.1107 8245121 PMC2119875

[B90] Wang M. Cao R. Zhang L. Yang X. Liu J. Xu M. (2020). Remdesivir and chloroquine effectively inhibit the recently emerged novel coronavirus (2019-nCoV) *in vitro* . Cell Res. 30 (3), 269–271. 10.1038/s41422-020-0282-0 32020029 PMC7054408

[B91] Webb R. R. (1955). Largactil in psychiatry. Med. J. Aust. 1, 759–761 14393177

[B92] Weiss S. R. Leibowitz J. L. (2011). Coronavirus pathogenesis. Adv. Virus Res. 81, 85–164. 10.1016/b978-0-12-385885-6.00009-2 22094080 PMC7149603

[B93] Weiss S. R. Navas-Martin S. (2005). Coronavirus pathogenesis and the emerging pathogen severe acute respiratory syndrome coronavirus. Microbiol. Mol. Biol. Rev. 69 (4), 635–664. 10.1128/MMBR.69.4.635-664 16339739 PMC1306801

[B94] Weston S. Coleman C. M. Haupt R. Logue J. Matthews K. Li Y. (2020). Broad Anti-coronavirus Activity of Food and Drug Administration-Approved Drugs against SARS-CoV-2 In Vitro and SARS-CoV In Vivo. J. Virol. 94 (21), e01218-20 10.1128/JVI.01218-20 32817221 PMC7565640

[B95] Wong S. S. Guan Y. Peiris J. S. Yuen K. Y. (2005). Characterization and complete genome sequence of a novel coronavirus, coronavirus HKU1, from patients with pneumonia. J. Virol. 79 (2), 884–895. 10.1128/JVI.79.2.884-895.2005 15613317 PMC538593

[B96] Woo P. C. Lau S. K. Chu C. M. Chan K. H. Tsoi H. W. Huang Y. (2005). Regional office for the western pacific, & seminar on Japanese encephalitis and other.

[B97] World Health Organization (1962). Final Report Seminar on Japanese encephalitis and other arbovirus infections, Tokyo, Japan 5–14 November 1962 . Tokyo, Japan.

[B98] Wu A. Peng Y. Huang B. Ding X. Wang X. Niu P. (2020). Genome composition and divergence of the novel coronavirus (2019-nCoV) originating in china. Cell Host Microbe 27, 325–328. 10.1016/j.chom.2020.02.001 32035028 PMC7154514

[B99] Wu F. Zhao S. Yu B. Chen Y.-M. Wang W. Song Z.-G. (2020). A new coronavirus associated with human respiratory disease in china. Nature 579 (7798), 265–269. 10.1038/s41586-020-2008-3 32015508 PMC7094943

[B100] Yang N. Shen H. M. (2020). Targeting the endocytic pathway and autophagy process as a novel therapeutic strategy in COVID-19. Int. J. Biol. Sci. 15 (10), 1724–1731. 10.7150/ijbs.45498.PMID:32226290 PMC7098027 32226290

[B101] Ye G. Pan Z. Pan Y. Deng Q. Chen L. Li J. (2020). Clinical characteristics of severe acute respiratory syndrome coronavirus 2 reactivation. J. Infect. 80 (5), e14–e17. 10.1016/j.jinf.2020.03.001 PMC7102560 32171867

[B102] Zaki A. M. van Boheemen S. Bestebroer T. M. Osterhaus A. D. Fouchier R. A. (2012). Isolation of a novel coronavirus from a man with pneumonia in Saudi Arabia. N. Engl. J. Med. 367 (19), 1814–1820. 10.1056/NEJMoa1211721 23075143

[B103] Zucker S. Zarrabi H. M. Schubach W. H. Varma A. Derman R. Lysik R. M. (1990). Chlorpromazine-induced immunopathy: progressive increase in serum IgM. Medicine 69 (2), 92–100. 10.1097/00005792-199069020-00003 2319941

