# Supplementary material for: The Large Action of Chlorpromazine: Translational and Transdisciplinary Considerations in the Face of COVID-19
Source: Front Pharmacol. 2020 Dec 16;11:577678. doi: 10.3389/fphar.2020.577678 (PMC7772402; doi:10.3389/fphar.2020.577678)
Supplement: Supplementary file 1 [file datasheet1.pdf]

PsyCHovid : CPZ Protocol for Covid-19 and Psychosis		
Phase 1 : Subjects Enrollment		
	Inclusion Criteria	Exclusion Criteria
1	Patients already treated for psychosis admitted to hospital.	History of severe hypersensitivity to CPZ or any other phenothiazine.
2	Age 18 and 65;<br>Hospitalized on an acute care ward due to COVID-19.	Active use of anti-viral therapies directed at SARS-CoV-2, except for remdesivir at the discretion of the treating physician. Antibiotics not directed at SARS-CoV-2 are allowed.
3	Gender: both males and females.	History of congenital long QT syndrome or known history of prolonged QT interval corrected by the Fridericia correction formula (QTcF) > 500 msec on electrocardiogram performed within 60 days of randomization.
4	Positive RT-PCR assay for SARS-CoV-2 on a nasopharyngeal swab sample.	Patients who have been hospitalized for COVID-19 for more than 72 hours, including the hospitalization time at another hospital for patients who were transferred.
5		Use of hydroxychloroquine, chloroquine or azithromycin within 30 days of Day 1.
6	The subject (or legally acceptable representative if applicable) must provide written informed consent for the trial.	Enrollment in another investigational study within 30 days of Day 1.
7		Known psychiatric or substance abuse disorder that would interfere with the requirements of the trial.
8		Unwilling or unable to comply with the study protocol.
9		Any condition, which in the opinion of the investigator, would preclude participation in the trial.
10		Healthy volunteers not allowed to participate.

Appendix. PsyCHovid : CPZ Protocol for Covid-19 and Psychosis

Phase 2 : Subjects Randomization (Two Groups)		Intervention\Treatment
Group A : **Placebo Arm**	Placebo Comparator: BSC<br>No active, only best supportive care (BSC).	Other: TAU
Group B : **Interventional Arm**	Experimental: CPZ+ BSC<br>Chlorpromazine tablets.	Drug: CPZ

Protocol Parameters	
Baseline :	Patients already treated for psychosis and receiving antipsychotic medication.
Interventional Protocol :	1- Add-on CPZ and decrease their current antipsychotic and sleep pills.<br>2- Randomization will be between two groups of trial; One will receive CPZ and decrease the antipsychotic and Experimental Oral Psychoses Adult: 25 mg at night 3 days then 50 mg 3 days and; may be given as a single 75 mg dose at night.
Comparaison group :	No change.
Treatment Variables :	CPZ, Antipsychotic medications, and doses.
Contraindications :	*Subjects with the following Conditions\Diseases*: Hypersensitivity to phenothiazine; Preexisting CNS Depression, Coma, Bone-Marrow Supression; Phaeochromocytoma; Lactation, History of Neuroleptic Malignant Syndrome.
Special Precautions :	*Subjects should be careful if they have the following Conditions\Diseases:* Parkinson's Disease; CV Disease; Renal or Hepatic Impairment; Jaundice; DM; Hypothyroidism; Paralytic ileus; Prostatic Hyperplasia or Urinary Retention; Epilepsy or History of Seizures; Myasthenia Gravis; Pregnancy;<br>*Subjects they should avoid:* direct sunlight.
Timeline :	Depends on the outcomes, if primary outcomes present on the subject, time frame will be: 15 days, if seconday outcomes present on the subject, time frame : will be through study completion/discharge (an average of 30 days with a maximum of 4 months.
Maintenance :	25-100 mg night increased to 300mg daily as required in psychotic patients (25-100mg tid). With a decrease of their usual night medication. The decrease of the current antipsychotics will be based on the Chlorpromazine equivalent table. For instance if a patient took 6 mg of risperidone, the CPZ equivalent is 300mg, so the adjusted dosage of risperidone after randomization to experimental CPZ harm at 100mg will be 4 mg.
For Research Purposes :	**CPZeq method is set in 300 mg/day of chlorpromazine as a maintenance dose.**<br><br>*The concept of CPZeq was derived from the potency for Dopamine receptor blockade, which was determined empirically to judge the dose equivalence between different Antipsychotic agents.*<br><br>-Shih-Ku LinLin, Shih-Ku, et al. "Comparison of the Defined Daily Dose and Chlorpromazine Equivalent Methods in Antipsychotic Drug Utilization in Six Asian Countries." (2018): 1847-1852.
Outcome Measures :	
**A. Primary Outcome Measures :**	**1. A composite endpoint of mortality.**<br>**2. Ongoing need for hospitalization.**<br>**3. Requirement for mechanical ventilation**/extracorporeal membrane oxygenation (ECMO) at Day 15 after randomization.<br>**-Time Frame :** 15 days.<br>- **Determine if :** CPZ + best supportive care (BSC) as compared to TAU + BSC reduces the composite endpoint of mortality, ongoing need for hospitalization, or requirement for mechanical ventilation/extracorporeal membrane oxygenation (ECMO) at Day 15 after randomization.

**B. Secondary Outcome Measures :**	**1. Time to Clinical Improvement.**<br>**-Time Frame :** Through study completion/discharge (an average of 30 days with a maximum of 4 months).<br>**-Determine if :** CPZ + BSC as compared to TAU + BSC reduces time to clinical improvement as defined by a decline of 2 categories or more from the baseline on the modified 7-category ordinal scale of clinical status of hospitalized influenza patients.
	**2. Inpatient mortality.**<br>**-Time Frame :** Through study completion/discharge (an average of 30 days with a maximum of 4 months).<br>**- Determine if :** CPZ + BSC as compared to TAU + BSC reduces inpatient mortality.
	**3. Duration of hospitalization.**<br>**-Time Frame :** Through study completion/discharge (an average of 30 days with a maximum of 4 months).<br>**-Determine if :** CPZ + BSC as compared to TAU + BSC shortens the duration of hospitalization.
	**4. Duration of intubation for mechanical ventilation.**<br>**-Time Frame :** Through study completion/discharge (an average of 30 days with a maximum of 4 months).<br>**-Determine if :** CPZ + BSC as compared to TAU + BSC shortens the duration of intubation for mechanical ventilation.
	**5. Time to normalization of temperature.**<br>- **Time Frame :** Through study completion/discharge (an average of 30 days with a maximum of 4 months).<br>-**Determine if :** CPZ + BSC as compared to TAU + BSC reduces the time to normalization of temperature (T < 37.5 for 48 hours).
	**6. Maximum severity of COVID19 illness.**<br>**-Time Frame :** Through study completion/discharge (an average of 30 days with a maximum of 4 months).<br>**-Determine if :** CPZ + BSC as compared to TAU + BSC reduces the maximum severity of COVID-19 illness based on the modified 7-category ordinal scale of clinical status of hospitalized influenza patients.<br>*(Score range 1-7, higher scores equals worse outcome).*

	**7. Biochemical Responses:**<br>*A. Airal load of SARS-CoV-2 on a nasopharyngeal sample.*<br>**-Time Frame:** day 7 from randomization.<br>*B. Serum viral load of SARS-CoV-2.*<br>**-Time Frame:** day: Day15.<br>C. *Biochemical response: C-reactive protein (CRP).*<br>**-Time Frame:** Days: 3,15.
	**8. Rates of Serious Adverse Events.**<br>**-Time Frame :** Days 7, 15.
	**9. Rates of Non-Serious Side Effects**.<br>-**Time Frame :** Days 7,15.
	**10. Extrapyramidal Assessment:** ESRS Scale.<br>-**Time Frame :** Days 7,15.
	**11. Subjective Cognitive Complains:** SSTICS.<br>**-Time Frame :** Days 7,15.
	**12. Prolactine Level.**<br>-**Time Frame :** Days 15, 30.
	**13. Psychiatric Symptoms Assessment: SSPI (Peter Liddle) Time : Days 7, 15, 30**
